# NLRP3 Inflammasome and Inflammatory Response in Aging Disorders: The Entanglement of Redox Modulation in Different Outcomes

**DOI:** 10.3390/cells14130994

**Published:** 2025-06-29

**Authors:** Bhavana Chhunchha, Eri Kubo, Deepali Lehri, Dhirendra P. Singh

**Affiliations:** 1 Department of Ophthalmology and Visual Sciences, University of Nebraska Medical Center, Omaha, NE 68198, USA; 2 Department of Ophthalmology, Kanazawa Medical University, Ishikawa 9200293, Japan

**Keywords:** oxidative stress, aging, peroxiredoxin 6, NF-*ĸ*B, NLRP3, TXNIP, Klf9, Caspase-1, IL-1β, Nrf2

## Abstract

Increasing evidence reveals that the deregulation of cellular antioxidant response with advancing age, resulting in the continuing amplification of oxidative stress-induced inflammatory response, is a pre-eminent cause for the onset of aging-related disease states, including blinding diseases. However, several safeguards, like an antioxidant defense system, are genetically in place to maintain redox homeostasis. Nonetheless, if the homeostatic capacity of such systems fails (like in aging), an inflammatory pathway elicited by excessive oxidative stress-evoked aberrant NLRP3 (NOD, LRR- and pyrin domain-containing protein 3) inflammasome activation can become pathogenic and lead to disease states. Among all known inflammasomes, NLRP3 is the most studied and acts as an intracellular sensor to detect danger(s). Upon activation, NLRP3 recruits apoptosis-associated speck-like protein containing a CARD (ASC) oligomerization and facilitates the recruitment of activated Caspase-1 (Cas-1), which results in the release of inflammatory cytokines, IL-1β and IL-18 and the activation of GasderminD, an executor of pyroptosis. NLRP3 inflammasome is tightly regulated in favor of cell health. However, when and how the activation of NLRP3 and its inflammatory components goes awry, leading to cellular derangement, and what regulatory factors are involved in the normal physiological and aging/oxidative conditions will be included in this review. Also, we address the latest findings to highlight the connection between oxidative stress, antioxidants, and NLRP3 activation as this begets aging diseases and explore the cellular pathways that are in place to regulate oxidative-induced inflammations and the pathobiological consequences of dysregulated inflammatory responses and vice versa.

## 1. Introduction

The NLR family protein NLRP3 inflammasome is a component of the inflammatory process that senses many pathogen-derived, environmental and host-derived factors, including reactive oxygen species (ROS) (oxidative stress) [1]. Upon activation, NLRP3 triggers the production of bioactive inflammatory cytokines and is pathogenic in complex diseases such as multiple sclerosis, type 2 diabetes, atherosclerosis, Alzheimer’s disease and many more disorders. ROS is implicated as an activator of NLRP3. ROS are generated through oxygen metabolism in mitochondria and cellular enzymes [2,3]. ROS function(s) is tightly regulated by a versatile antioxidant defense system that fine-tunes intracellular ROS levels in favor of cell health. Nevertheless, in response to stressors, ROS concentrations overcome the antioxidant capacity and thereby generate increasing levels of oxidative stress-induced inflammatory response. Recent emerging studies accentuate that oxidative stress plays an obligatory role in the activation of inflammasomes, particularly, the NLRP3 inflammasome [4,5,6,7]. However, the exact role of ROS in the activation of the NLRP3 inflammasome is not well understood. Specifically, how ROS activates the NLRP3 inflammasome-mediated inflammatory pathway is not well understood. Nonetheless, studies have shown that the inhibition of ROS can abate NLRP3 activation, suggesting the prime role of ROS in NLRP3 activation and regulation [8]. In this scenario, we think that NLRP3 or its inducers, like ROS, can be an attractive vital therapeutic target for inhibitors/drugs [6,7,9,10].

Recently, NLRP3 expression/activation-mediated inflammation has been identified in non-myeloid cells, like endothelial and epithelial cells [6,11,12,13,14,15,16,17,18,19]. Generally, inflammasomes are cytoplasmic multiprotein complexes and are pivotal molecules for inflammatory signaling and cell death [20]. In a resting state, NLRP3 exists as an inactive ‘cage’ (multiprotein complex) and, upon activation in response to the provocation of various stimuli, it recruits the activated form of the inflammasome inflammatory signaling complex and components [6,21,22]. The inflammasome complex leads to the formation of an apoptosis-associated speck-like protein containing CARD (ASC). Mostly, the inflammasome(s) (NLRP3) regulates the activation and discharge of pro-inflammatory cytokines, IL-1β and IL-18, generated in cytosol, including many damage-associated endogenous inflammatory components (alarmins) released during pyroptosis [5,23]. The transition from the inactive NLRP3 inflammasome cage to the activated inflammasome requires major structural changes/reorganization that is modulated via Toll-like receptor (TLR) ligands with integrated priming along with activation signals related to cellular stresses. However, the exact activity of these activating signals are still ambiguous and warrant investigation, but they are involved in the aberrant NLRP3 regulation of post-translational modification, subcellular expression patterns with major conformational changes in NLRP3 (via ADP to ATP exchange), and the recruitment of inflammasome-pro-inflammatory components, such as the adaptor protein, ASC, the effector, Caspase-1 (Cas-1) and the executor of pyroptotic cell death, GasderminD (GSDMD). ASC is an adaptor molecule and is recruited by the NLRP3 inflammasome. The oligomerization of the recruited ASC results in the formation of ASC specks, which can be visualized by even light microscopy and flow cytometry [14,24].

In aging, the decline in a cell’s protective functions is an inevitable natural biological phenomenon marked by a decline in cellular antioxidant defenses and increased ROS accumulation [25,26]. The excessive production of ROS (beyond the threshold physiological levels) is a crucial factor in triggering the NLRP3 inflammasome activation pathway. Moreover, when cells/tissues face an adverse state within the cellular microenvironment, mitochondria disintegration results in a further increase in ROS levels, resulting in NLRP3 inflammasome aberrant activation [6,27,28,29,30]. However, to limit the injurious process and to maintain threshold levels of survival redox signaling, cells have inheritably evolved to have a nuclear factor erythroid 2-related factor 2 (Nrf2) antioxidant defense mechanism, which regulates and activates a series of antioxidants like superoxide dismutase (SOD), glutathione peroxidases (GPxs), thioredoxin (TRX), catalase (CAT), and peroxiredoxins (Prdxs), such as the selenium (Sel)-independent Prdx6 (a Sel facilitator for selenoproteins, like Gpx4) [31], including a TRX and TRX-interacting protein (TXNIP) redox system to mitigate oxidative and phospholipid peroxidase stresses, thereby controlling cellular and mitochondrial redox homeostasis [6,28,31,32,33,34,35,36,37]. TXNIP is a TRX binding protein and acts as inhibitor for the cytosolic antioxidant TRX and mitochondrial TRX (activated form) and, in this way, regulates cellular redox balance [37]. However, the magnitude of TXNIP-TRX crosstalk (interaction) is considered a critical event for TXNIP-NLRP3 interaction and NLRP3 inflammasome activation [37,38]. Under the excessive oxidative conditions, TXNIP is released from TRX and activates inflammatory signaling via activating the NLRP3 inflammasome [39]. Studies have shown that aberrant TXNIP expression causes increased NLRP3 inflammasome activation and pyroptosis [40,41,42,43,44,45,46,47,48,49]. One key pathway linked to oxidative stress and inflammation is the activation of redox-dependent transcription factors such as the nuclear factor kappa-light-chain-enhancer of activated B cells (NF-*κ*B) and activator protein 1 (AP-1) via mitogen-activated protein kinases (MAP kinases), including transcription factor Kruppel-like factor 9 (Klf9) [48,50,51]. These factors regulate the production of NLRP3 and pro-inflammatory cytokines during oxidative stress [52,53,54,55]. Studies clearly point out that reducing ROS production by means of antioxidant supply can potentially inhibit inflammasome activation and pyroptosis [6,56].

In this review, we summarize current knowledge on the molecular mechanisms underlying in the regulation and activation of the NLRP3 inflammasome(s) and its background, with particular emphasis on the role of ROS-driven oxidative stress in the aberrant activation of the NLRP3 inflammasome and its components. We also discuss the intricate relationship between oxidative stress, antioxidants, and inflammasome activation in age-related diseases. Additionally, we include the contribution(s) of prime redox-active transcription factors in the modulation of the antioxidant system and NLRP3 inflammasome pathways. We consider that this review, bearing current findings along with an analysis of the published literature, will deliver a comprehensive picture of the activation and regulation of NLRP3 under physiological and aberrant redox cellular conditions.

## 2. Inflammasomes

### 2.1. Overview: Inflammasomes and Their Discovery

**Figure 1:**
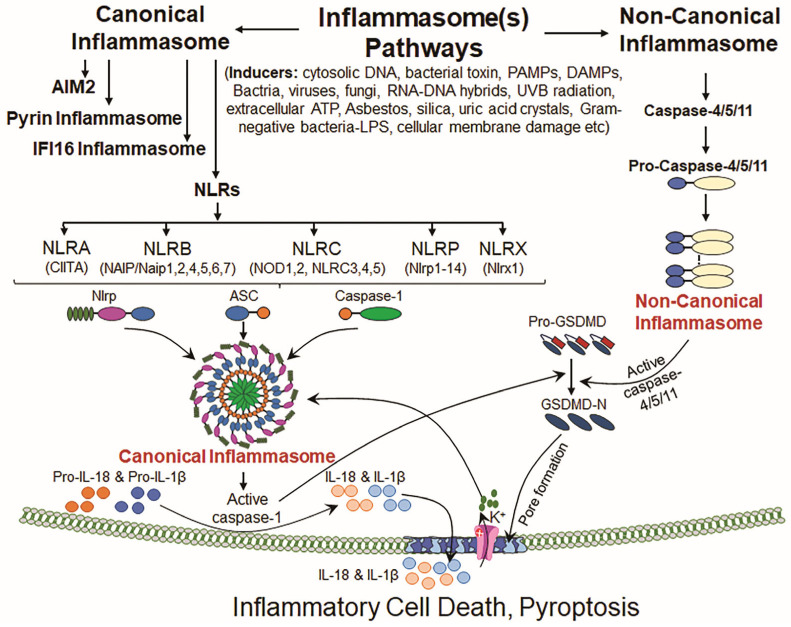
Canonical and non-canonical inflammasome signaling pathways. Inflammasome signaling is categorized into canonical and non-canonical signaling pathways based upon the activation of types of caspase(s) involved in the biological phenomenon. The canonical inflammasome NLR family consists of the following prime members: NLRA, NLRB, NLRC, NLRP and NLRX and AIM2, Pyrin and IFI16 inflammasomes. These inflammasomes are activated by various cellular and external stimuli/inducers, resulting in NLRP priming, which increases NLRP transcription and pro-Cas-1 synthesis. Upon activation, NLRP inflammasome assembly formation occurs, involving pro-Cas-1 and ASC protein. Canonical inflammasome assembly activation results in bioactive Cas-1, which is subsequently processed and releases IL-1β and IL-18. Activated Cas-1 also cleaves GSDMD at its N-terminal domain (bioactive fragment), facilitating pore formation in the plasma membrane. These pores enable the release of IL-1β and IL-18, triggering pyroptosis. In non-canonical pathways, pro-Cas-4/5 (in human) or pro-Cas-11 (in mice) is activated by inducers such as Gram-negative bacteria or by cellular membrane damage and forms non-canonical inflammasome assembly, resulting in activated Cas-4/5/11. Bioactive Cas-4/5/11 activates GSDMD by cleaving it, and the bioactive N-terminal domain enables pore formation in the plasma membrane and thereby allows K^+^ influx, which further leads to the activation of the NLRP inflammasome protein complex. Upon NLRP3 inflammasome activation, the release of bioactive IL-1β and IL-18 in the presence of bioactive Cas-1 induces the inflammatory programmed cell death.

The word “Inflammasome” was first used by Martinon et al. 2002 [57,58] to illustrate an intracellular multiprotein complex of high molecular weight that acts as a molecular stage in cells that activates inflammatory caspases (Cas) (cysteine-dependent aspartate-specific proteases), previously known as IL (interleukin)-converting enzymes, and is responsible for the maturation of IL-1β or other ILs [57,59]. Inflammasomes mostly contain a sensor protein, the adaptor protein ASC, and Cas-1 [60,61] but the participating components may depend upon the activating types of stimuli. However, the acute inflammatory response evoked via inflammasome activation plays an important role(s) in the host’s defense, like the elimination or neutralization of infections and wound healing caused by trauma or other types of tissue damage, while inflammasome-mediated chronic inflammatory response (inflammaging) induced via metabolites released from dead cells (like apoptosis), which act as a chronic stimulator and lead to the onset of aging diseases [62,63,64]. Furthermore, the inflammasomes pathway is provoked and activated when pattern recognition receptors (PRRs), for instance, the nucleotide-binding domain leucine-rich repeat receptor (NLR) and AIM2-like receptors, perceive the presence of PAMPs or DAMPs that are released and/or exist in cytosol. This aberrant process results in pyroptosis. Pyroptosis also occurs when cells are exposed to microbial pathogens [65]. TLRs, specifically, recognize microbial metabolites and contribute to adaptive immunity through downstream signaling involving NLRs [66]. Mechanistically, activated inflammasomes contain a sensor protein that oligomerizes with the adaptor molecule ASC. ASC undergoes autoproteolysis after oligomerization, which results in the cleavage of procaspase into its active form, Cas-1. Activated Cas-1 then acts on pro-interleukin-1β (pro-IL-1β) and pro- IL-18, and this event results in the existence of their biologically active forms. Further, activated Cas-1 activates the executor of pyroptosis, GSDMD, by cleaving it at its N-terminal domain, thereby leading to GSDMD-mediated pore formation in plasma membrane and the release of these cytokines and triggering pyroptosis (Figure 1) [67].

### 2.2. Distinct Members of the Inflammasome Family

An inflammasome is generally classified according to the identity of its sensor proteins, NLRs. The NLR family represents one of the most well-characterized inflammasomes and has been proposed to be a sensor for inflammasome complexes [68,69]. In both human and mice, multiple NLR family members have been identified. However, a total of 22 NLRs have been documented in humans, and components of the inflammasome complex and these sensing receptors lead to an array of cellular signaling, depending upon the nature of the stimuli [70,71,72]. Moreover, to date, five subfamilies have been identified and constitute the NLR family, including NLRA (CIITA and Ciita), NLRB (NAIP and Naip1,2,4,5,6,7), NLRC (NOD1, NOD2, NLRC3, NLRC4, and NLRC5), NLRP (NLRP1-NLRP14) and NLRX (NLRX1 and NLRx1). In addition to this is the AIM2 (PYHIN, pyrin and HIN domain-containing protein) family; AIM2 senses foreign double-stranded DNA and plays a role in inflammasome activation [73,74]. The ASC adaptor protein, encoded by the *Pycard* gene, is essential for assembling the inflammasome signaling complex [75]. Caspases, a family of aspartate-specific cysteine proteases, are involved in inflammation and apoptosis. Among them, Cas-1, -4, and -5 drive inflammatory responses. Cas-1 is found to play a central role in the activation of IL-1β [76]. Upon activation by DAMPs or PAMPs or viruses and/or oxidative inducers, this cascade results in inflammatory responses and cell death, pyroptosis [4,11,77,78,79]. Furthermore, inflammasome activation is implicated in protecting against internal/external stressors [80]. Conversely, the chronic abnormal activation of inflammasomes causes inflammatory reaction-driven metabolic diseases, including cancer, cardiovascular diseases, diabetes, ocular diseases, and neurodegenerative diseases [17,67,81,82,83,84]. Various sensor molecules, primarily from the NLR family, act as inflammasome initiators. Structurally, NLR proteins belong to the ATPase superfamily and contain three key domains: a caspase activation and recruitment domain (CARD) or Pyrin domain at the N-terminus, a nucleotide-binding and oligomerization domain (NACHT domain), and multiple leucine-rich repeats (LRRs) at their carboxy terminus [57,85]. To date, 14 NLRP family members (NLRP1-NLRP14) have been identified, each contributing uniquely to inflammasome formation and the regulation of the inflammatory pathway (Figure 1). As noted in the introductory statements, the NLRP3 inflammasome is well studied, and it has been documented that this inflammasome plays an unquestionable role in the initiation and development of many aging disorders, and it has been proposed that NLRP3 is a sensor for cellular organelle stress [86,87,88]. In this review, we mainly describe oxidative stress/aging and the NLRP3 inflammasome, including its activation and crosstalk/interactions and pathways, converging to its aberrant activation-mediated inflammatory responses. Nonetheless, we will also describe other important members of the inflammasome family in brief as enumerated herein.

### 2.3. NLRP1 Inflammasome

NLRP1 (NLR family Pyrin domain-containing 1) was the first NLR reported to form an inflammasome assembly. This inflammasome consists of multiple domains including NACHT, LRR (leucine-rich repeat), FIIND, CARD, and PYD (Pyrin domain). A single NLRP1 protein is reported in humans, while mice bear multiple paralogs—NLRP1A, NLRP1B, and NLRP1C. They share similar domain structures but are devoid of the Pyrin-like domain [57,89]. The NLRP1 inflammasome is suggested to be involved in neuroinflammation and brain aging, including neurodegenerative diseases like Alzheimer’s disease [90,91]. Furthermore, NLRP1 plays a critical role in inflammasome activation in neurons, leading to the genesis and release of pro-inflammatory cytokines, for example, IL-1β and IL-18. The release of these cytokines can contribute to neuroinflammation and may cause age-related cognitive decline [90,92]. In addition to its role in the central nervous system, NLRP1 is suggested to play a role in the development of ocular diseases. In the corneal epithelium, it serves as a critical innate immune sensor, and its aberrant upregulation is associated with inflammation-regulated cell death and corneal insult/injury. Inhibiting NLRP1 signaling has been shown to protect human corneal epithelial cells from inflammation-linked cell death (Figure 1) [93,94].

### 2.4. NLRP6 Inflammasome

NLRP6 was originally known as PYPAF5 (PYRIN-containing Apaf-1-like protein 5) and belongs to the NLR family that patrols the cytosol of cells to detect pathogens and DAMPs and thus acts as a cytosolic sensor [70,95]. Similarly to NLRs and Toll-like receptors (TLRs), NLRP6 functions as a novel regulator of Cas-1 and NF-*κ*B activation and plays a crucial role in gut immunity or metabolic homeostasis [70,96]. NLRP6 expression is tightly regulated at both transcriptional and post-translational levels in response to upstream pathogenic and metabolic stressors. Upon activation, NLRP6 oligomerizes with ASC and Cas-1 to form an inflammasome complex and thereby activates pro-inflammatory proteins IL-1β and IL-18. The dysregulation of NLRP6 inflammasome activity has been linked to intestinal disorders, metabolic diseases, and inflammatory pathologies, highlighting its relevant contribution in maintaining cellular signaling and balancing cell physiology (Figure 1) [97,98]. However, the emerging literature proposes the cell type-specific activity of NLRP6 with a vital contribution to cell/tissue defense in response to microbes and gut inflammation [4,95,99,100].

### 2.5. The NAIP–NLRC4 Inflammasome

Formerly, the NLR family caspase activation and recruitment domain-containing protein was known as IPAF (ICE protease-activating factor), but now, it is recognized as a cytosolic sensor for bacterial components, particularly flagellin and the type III secretion system (T3SS), and is named NLRC4 [101,102]. The NLRC4 inflammasome is a vital sensing machine, which directly senses microbial infection and response to cellular stimulus during sustained chronic inflammation, even in the absence of pathogens. Regardless of its well-described role in host immune response, a complete definition of the molecular mechanisms in NLRC4 inflammasome genesis in humans is not clear yet. However, recently, it was documented that the human NAIP (neuronal apoptosis inhibitory protein) and NLRC4 employ peculiar mechanisms of activation. Unlike NLRC4, NAIP does not bear a receptor surface. It connects to triggering agents and integrates with the lasso-like motif and then cooperatively activates NAIP [103]. The NAIP-NLRC4 inflammasome plays a critical role in bacterial defense mechanisms and contributes largely to autoimmune and inflammatory responses, causing a disease state in a chronic condition. Further, distinct from other NLR inflammasomes, NLRC4 does not directly interact with its activators but depends upon on NAIP family members to recognize microbial ligands, triggering the formation of inflammasome assembly [67,104]. Upon activation, like other inflammasomes, the NLRC4 inflammasome recruits Cas-1, leading to the bioactivation of IL-1β into its active state and the initiation of inflammatory cell death, pyroptosis [104,105,106]. The expression of NLRC4 is upregulated in response to pro-inflammatory stimuli like genotoxic stresses, including tumor necrosis factor-alpha (TNFα) and transcription factor p53. The aberrant chronic activation of NLRC4 has been linked to bacterial infections, chronic inflammation, and inflammatory diseases of the gut and lungs (Figure 1) [107,108].

### 2.6. AIM2 Inflammasome

Absent in Melanoma 2 (AIM2) was first discovered as a gene that was not present in melanoma cell lines using subtractive cDNA hybridization [109]. AIM2 is an interferon-inducible gene and belongs to the PYHIN protein family. Recent studies reveal that the PYD of AIM2 self-oligomerize to trigger activation [110]. In normal situations, AIM2-mediated effector inflammatory cytokines and inflammatory cell death play an important role for host protection in response to bacterial/viral infection [111]; however, its overactivation becomes detrimental in several chronic inflammatory diseases [112,113]. In homeostatic conditions, the sensing of self-DNA by AIM2 is modulated via a conserved mechanism that has evolved to categorize self-DNA found in the nucleus or degrade mislocalized DNA [114]. It is worth mentioning that DNA sequences, like the TTAGGG repeat, which is found in mammalian telomere, inhibits the activation of the AIM2 inflammasome-mediated inflammatory pathway [115]. Interestingly, inhibitory ssDNA (oligodeoxynucleotide) [116] containing four repeats of TTAGGG has been found to inhibit AIM2 signaling by interfering with activating DNA ligands [115]; however the regulation of this process requires validation in vivo. Furthermore, AIM2 resides in cytosol and acts as a cytosolic sensor of bacterial/viral and cellular erratic double-stranded DNA (dsDNA). AIM2 adheres to DNA via its HIN200 domain, activating inflammasome assembly and subsequently initiating inflammatory responses [117]. Activated AIM2 inflammasomes play a crucial part in chronic inflammation, and it is reported that probenecid mitigates chronic heart failure by attenuating the inflammation processes [118]. Moreover, AIM2 belongs to the AIM2-like receptor (ALR) family and contains four members in the human genome. It has been shown that once AIM2 is activated in response to genotoxic stimulus, it recruits ASC containing a CARD, promoting the binding of Cas-1 and AIM2 inflammasome formation. Interestingly, this mechanism allows AIM2 to provide defense against viral and bacterial DNA, and this process is fully independent of NLRP3, interferons, or TLR signaling pathways [119]. In the face of microbial infections, AIM2 inflammasome activation in response to dsDNA damage triggers the production of pro-inflammatory cytokines and GSDMD-mediated inflammatory programmed cell death—pyroptosis [120]. In this context, similar to the NLRP3 inflammasome, AIM2 activation initiates the formation of the inflammasome complex and activates inflammatory caspases, leading to the maturation and secretion of cytokines IL-1β and IL-18, thereby initiating pro-inflammatory cell death (Figure 1) [121]. However, the dsDNA-evoked activation of the AIM2 inflammasome not only drives Cas-1-dependent pyroptotic cell death but also can provoke Cas-8-mediated apoptosis, depending upon the magnitude of dsDNA. It is observed that a higher amount of infected DNA causes the occurrence of pyroptosis [122].

### 2.7. IFI16 Inflammasome

Interferon gamma-inducible protein 16 (IFI16) and AIM2 inflammasome assemblies or complexes are different to the aforementioned NLR-dependent inflammasomes, as both IFI16 and AIM2 specifically sense cytosolic DNA through their hematopoietic interferon-inducible nuclear (HIN) domain/motif, and they miss the NOD required for self-oligomerization. In this regard, the electrostatic interaction of a double-stranded DNA backbone with a positively charged HIN domain allows AIM2 autoinhibition [123] and then leads to the formation of a polymerization platform for ASC, thereby underpinning an inflammasome complex [124,125]. Dissimilar to Aim2, the HIN domain of IFI16 has a comparatively lower affinity to DNA-binding, and the PYD (the non-DNA binding) of IGI16 is requisite for the cooperative assembly of IFI16 strings on DNA [126]. Moreover, the IFI16 gene belongs to the HIN200 family and acts as a sensor for nuclear and cytosolic DNA. It has been identified as a target of interferons (IFNs) and bears an HIN200 ligand-binding domain and a PYRIN domain. This configuration allows it to play a part in apoptosis and inflammasome formation [127]. However, similar to AIM2, IFI16 detects cytosolic dsDNA, which makes it capable of detecting nuclear pathogens, such as herpesviruses, including Kaposi’s Sarcoma-associated herpesvirus (KSHV) [128]. IFI16 also functions as a sensor for nuclear pathogens, triggering inflammasome formation. Upon recognizing viral DNA, IFI16 binds to ASC and pro-Cas-1 to constitute a bioactive form of inflammasome assembly and its components, triggering inflammatory cytokine release [129]. Additionally, oxidative stress induced by H_2_O_2_ upregulates IFI16 gene expression, leading to the activation of p53 transcriptional activity in endothelial cells. This suggests a broader role for IFI16 in DNA damage response and cell cycle regulation beyond its immune functions (Figure 1) [127].

### 2.8. Pyrin Inflammasome

Pyrin, also known as marenostrin or TRIM20, is a high-molecular-mass (~86 kDa) sensor protein mostly expressed in immune cells such as dendritic cells, monocytes, eosinophils, and granulocytes [130,131]. It is encoded by the MEFV gene and responds to many inflammatory signals such as interferon gamma (IFN-γ), lipopolysaccharide (LPS), TNF-α, IL-4, and IL-10 [132,133,134,135]. The activation of Pyrin inflammasome assembly occurs in response to microbial toxins and is further stimulated through cytokine-related signaling [131]. The Pyrin protein bears five distinct functional domains, including a PYD domain that interacts with the inflammatory adaptor protein ASC, which in turn leads to Cas-1 activation and the Cas-1-mediated release and secretion of bioactive IL-1β [130]. Studies have shown that mutations in Pyrin’s B30.2 domain result in autoinflammation-evoked diseases, which are related to the increased production of IL-1β due to the overactivation of Cas-1 [136]. Further, the N-terminal PYD domain also interacts with NF-*ĸ*B, and NF-*ĸ*B is an activator of inflammasomes and inflammatory cytokines, leading to an aggressive inflammatory response. Also, Pyrin can facilitate the Cas-1 inflammasome cage in an ASC-dependent way after sensing an inactivating modification of the RhoA GTPase by microbes [137,138,139]. Pyrin recognizes the severity of pathogens through the cytoskeleton rearrangement of cells rather than bacterial toxins [140]. Studies have dictated that the C-terminal B30.2 domain is a possible cause of familial Mediterranean fever (FMF), a genetic autoinflammatory disorder [139,141]. Nonetheless, Pyrin inflammasome signaling can prevent dysbiosis and increase intestinal barrier integrity and can abate inflammation and tumorigenesis [142]. The oligomerization of Pyrin involves the B-box and α-helical coiled-coil domains, which bind to the cytoskeleton organization protein PSTPIP1/CS2BP1 [143,144]. In addition, Pyrin, like other types of inflammasomes, such as IFI16 and NLRP6, can detect specific danger signals from hosts/microorganisms and trigger the formation of the active inflammasome complex via the activation of Cas-1 (Figure 1).

## 3. NLRP3 Inflammasome and Function

The timely and instant sensing of injurious signaling within a cellular microenvironment and the loss of cell/tissue homeostasis and integrity, and thus subsequent responses against this aberrant event(s), is an important task of the cellular homeostatic system. Genetically destined pattern recognition receptors (PRRs) achieve this task by recognizing the damaging/pathogenic signals triggered by dangerous inductors like DAMPs/PAMPs, including microbes and oxidative stress inducers, and (PRRs) waking up the defense system when facing the onset of cell/tissue injuries, and they initiate the inflammatory pathway to defend cells [4,9,11,145]. However, inflammatory signaling is genetically dependent and produces vital biological responses to any tissue injury, crucial for tissue repair and body health. Acute and chronic or sterile inflammation induced by an imbalance of pro- and anti-inflammatory cytokines can cause tissue damage or vice versa. In this regard, a multi-protein assembly called an inflammasome such as NLRP3, NLRP6, NLRC4 and AIM2 has a pivotal role in triggering and sustaining inflammatory responses. Upon activation, the inflammasome and its mediated inflammatory pathway lead to a type of programmed cell death defined as pyroptosis, distinct from apoptosis [146].

Studies have shown that NLRP3 is present in multiple organelles; however, the contributions of different organelles to NLRP3 activation remain unclear. In resting conditions, NLRP3 is localized in cytosol, ER, mitochondria and the nucleus [147,148,149,150]. Various studies document that when activated, NLRP3 inflammasome formation occurs at the aforementioned organelle site, such as mitochondria, mitochondria-associated ER membranes, [147,150,151], lysosome membranes [86,152], and endosomes [86,87,153], including centrosomes [154,155]. Further, the NLRP3 inflammasome is present in both human and mouse genomes and is located on Chromosome 1. This gene encodes a protein called Cryopyrin, which is highly sensitive to ROS and can be activated by several stimuli, such as UVB radiation, extracellular ATP, asbestos, silica, and uric acid crystals [67,156,157,158]. Increased oxidative stress and NLRP3 activation, leading to an inflammatory response, has been documented in the development of several age-related disorders [159]. Aging cells, which produce increased levels of ROS due to the reduced expression and activity of antioxidants, are highly vulnerable to NLRP3-mediated inflammation, dependent cellular damage via the pyroptotic phenomenon [5,44,160,161]. As mentioned in the earlier section, upon activation, NLRP3 recruits the adaptor protein ASC and subsequently engages Cas-1, leading to the production of bioactive IL-1β and IL-18. Concurrently, the activated Cas-1 cleaves GSDMD, releasing its active N-terminal domain. This event leads to the formation of lytic pores in cell membranes and the release of bioactive IL-1β and IL-18, which results in inflammatory programmed cell death, pyroptosis [5].

### 3.1. First Signal: Priming

**Figure 2:**
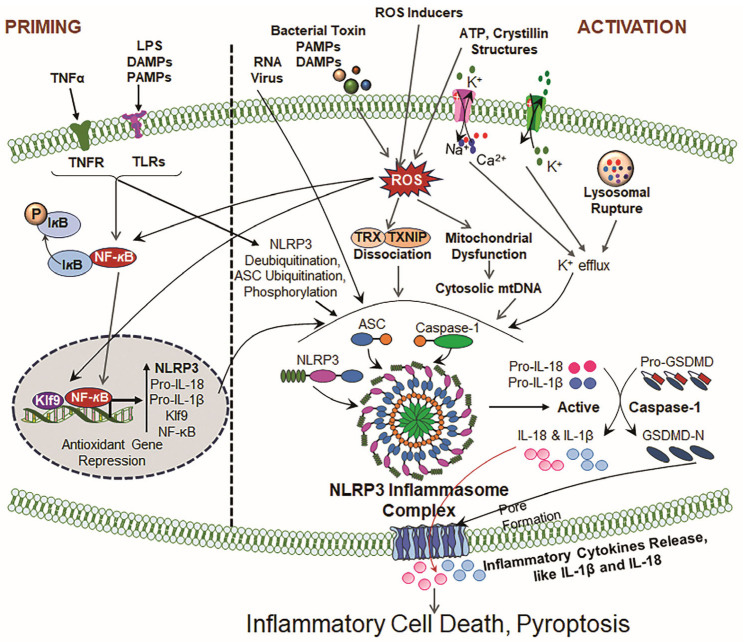
Illustrative cartoons of NLRP3 priming and activation signaling pathways. The NLRP3 inflammasome activation process contains two prime signals. Signal 1 (Priming): Driven by TLRs and PRRs in response to LPS, tumor necrosis factor receptor (TNFR) and ROS. This event(s) results in the upregulation of NLRP3, pro-IL-1β, and pro-IL-18 transcription via NF-*κ*B activation and other transcription factors and the suppression of antioxidant genes, like Prdx6 (by Klf9 [6,28,51]). Signal 2 (Activation): Induced by ATP, crystallin structures, for instance, uric acid, silica, and cholesterol crystals, and mitochondrial dysfunction/disintegration. The above-noted inducers cause events, including ROS generation, ion fluxes (K^+^ efflux, Ca^2+^ influx) and lysosomal rupture, that provoke NLRP3 oligomerization and the recruitment of ASC, leading to the formation of activated NLRP3 inflammasome assembly, and facilitate the activation of Cas-1 and bioactive IL-1β and IL-18. These consequences finally result in GSDMD activation via Cas-1 and mean that the bioactive N-terminal GSDMD forms membrane pores, leading to the release of bioactive IL-1β and IL-18, cell swelling and inflammatory cell death (pyroptosis).

Based on the current literature, we ideate that NLRP3 inflammasome activation occurs by a wide array of stimuli and that NLRP3 inflammasome activators (most of them) have not been revealed to directly bind to NLRP3. Therefore, it is anticipated that NLRP3 only senses aberrant cellular damage and adverse changes caused by the inducers/activators. The adverse changes in cells can be stimulated by K^+^ efflux, the lysosomal dysfunction-induced release of cathepsin B, the generation of increased ROS due to mitochondrial and endoplasmic reticulum dysregulation [60,61,162]. Nonetheless, recent reports have dictated that K^+^ efflux is not necessary for the activation of the NLRP3 inflammasome [163]. Furthermore, the activation of NLRP3 needs a priming signal in addition to various activators [53]. The priming step is critical for NLRP3 activation. It involves the activation of the transcription factor NF-*κ*B, and this may require ROS induction. Upon activation, NF-*κ*B translocates to the nucleus and thereby activates the NLRP3, pro-IL-1β, and other inflammasome components by binding to its response elements present in their promoters [164]. In an inactive state, the low level of NF-*κ*B leads to the insufficient expression of pro-IL-1β, thus preventing NLRP3 activation [23,27,159,165]. During priming, levels of pro-Cas-1, pro-IL-18, and ASC remain unchanged. Ensuing priming, NF-*κ*B get activated via binding to the caspase-IKK complex, promoting the transcription and translocation of NF-*κ*B [166]. The transcriptional role of priming in NLRP3 activation involves the upregulation of NLRP3 and pro-IL-1β [53]. Recent studies demonstrate the pivotal role of signaling molecules like caspase-8 and FADD in evoking NLRP3 during priming signaling [167,168]. Moreover, even a short exposure of LPS rapidly promotes the activation of the NLRP3 inflammasome [164,169]. Further, the LPS-induced phosphorylation of IRAK-1 dramatically increases NLRP3 inflammasome activation, independent of the IKK complex. This suggests that IRAK-1 participates in inflammasome activation, even in the absence of NF-κB signaling [170]. It is observed that during the priming process, the LRR domain of NLRP3 undergoes ubiquitination, which is reversed by the deubiquitinating enzyme BRCC3 (also called BRCC36). This deubiquitylation leads to an increase in NLRP3 inflammasome activation [164,171]. Recently, it has been suggested that NLRP3 phosphorylation during priming is an important event for its self-association and activation. Also, mitochondrial DNA synthesis, triggered by the activation of the IRF1 transcription factor, is necessary for NLRP3 inflammasome activation [172]. Recently, our group has identified that Klf9, a transcription factor, plays an important role in the expression and activation of NLRP3 by binding to its response element present in the NLRP3 gene promoter [6]. We have shown that both NF-kB and Klf9 are essential for the peak upregulation of NLRP3 transcription and activation under the condition of oxidative stress. However, the first priming signal controls NLRP3 activation through both transcription-dependent and -independent mechanisms (Figure 2).

### 3.2. Second Signal: Activation

Following the priming phase, NLRP3 activation can be triggered by a wide range of stimuli, including extracellular ATP, changes in potassium ion concentrations, ionophores, heme, particulate matter and various bacterial toxins such as nigericin, LPS and pathogen-associated RNA/DNA [60,173,174]. A cascade of events occur that leads to the inflammasome complex and its activation in response to the above-mentioned activator(s) [105,175,176]. However, key activators of the NLRP3 inflammasome include ATP and certain bacterial toxins, endogenous toxins, and microbial products [174,177]. Noteworthy is that TLR ligands do not directly participate in the activation of the NLRP3 inflammasome and its mediated pathway, but they play a vital role in the priming step of activation. Moreover, ATP or bacterial toxins are required for NLRP3 inflammasome activation in certain types of cells [53,54,178]. A two-signal model describes the activation of NLRP3 in macrophages: priming is triggered by endogenous molecules or microbial toxins that induce NLRP3 expression and pro-IL-1β through NF-*κ*B activation, and then activation occurs by various stimulants like ATP, exogenous molecules (e.g., particulate matter, silica), UVB irradiation, viral RNA, and pore-forming toxins (Figure 2). Recent studies have shown several other factors/pathways, including complement, protein kinase R (PKR), purine receptor signaling, necroptosis and ZBP1 signaling, are involved in NLRP3 inflammasome activation [179,180].

## 4. ROS-Driven Oxidative Stress and Inflammatory Cell Death, Pyroptosis

Increased oxidative stress in response to the excessive production of ROS due to the dysregulation of antioxidant defense causes oxidative damage to proteins, DNA and membrane lipids and is considered to be a major culprit in different types of cell death, such as apoptosis, necrosis, necroptosis and pyroptosis [4,6,27,28,44,181,182,183,184]. Interestingly, crosstalk among the aforementioned death types has recently been identified. Dying cells or cells going to die in response to oxidative stressors, such as apoptotic or necrotic or necroptotic cells and cells under ferroptosis, are found to release various metabolites, including DAMPs or PAMPs, which trigger an inflammatory form of cell death, pyroptosis [63,185]. Pyroptosis has been recently characterized as a form of regulated cell death, which is different to other known cell death mechanisms like apoptosis and necrosis or necroptosis due to its distinct characteristics and regulatory pathways [186,187]. Unlike apoptosis, which encompasses the controlled degradation of cellular organelles/constituents, pyroptosis is triggered by inflammatory signals and results in the release of pro-inflammatory cytokines [188,189]. Both canonical and noncanonical inflammasomes recruit caspases for the activation of the inflammasome-mediated inflammatory pathway. The canonical pathway activation mostly engages Cas-1, while non-canonical inflammasome signaling involves Cas-4, Cas-5 (in human), and Cas-11 (in mice), to trigger pyroptosis. Furthermore, Gasdermins, particularly GSDMD and DFNA5, are critical players in pyroptosis [190,191]. While most members of the Gasdermin family are implicated in pyroptosis, Pejvakin (PJVK) is an exception and has not shown any pyroptotic functions to date. It is worth mentioning that the specific identity of the upstream molecular cascade responsible for ROS amplification and NLRP3 activation in aging is unclear and warrants active investigation. In addition, the exact mechanism(s) involved in how ROS activate Gasdermin, an executor of pyroptosis, is also not well understood and is an area of active research. Nonetheless ongoing research highlights that there is an intricate interplay between oxidative stress pathways and inflammatory responses. Based upon published reports, including our own group [6,63,64,185], oxidative stress serves as a critical regulator in pyroptosis, influencing both inflammasome activation and Gasdermin-dependent inflammatory cell death mechanisms [4,192,193]. Nevertheless, further work is needed to understand the crosstalk and interactions of the above-mentioned molecules, which may lead to the need to create a new and novel therapeutic strategy for diseases associated with dysregulated survival pathways in response to oxidative/aging stress. However, inflammasome-mediated inflammatory cell death can occur either via the canonical or non-canonical pathway, which is covered briefly below.

### 4.1. Canonical Pathway

So far, various extra-and intra-cellular stimuli such as bacteria, viruses, toxins, and oxidative stress, which can induce DNA/protein damage, have been identified, which activate inflammasome-sensing proteins like Nod-like receptors (NLRPs) and inflammasome-driven inflammatory cell death, pyroptosis [89,194]. NLR family members, including NLRA, NLRB, NLRC, NLRP and NLRX, detect different types of stimuli and provoke the formation of inflammasomes. Among all NLR and NLRP family members, NLRP3 is specifically sensitive to the changes in cellular homeostasis, including potassium efflux and cellular redox imbalance. Upon activation, the NLRP3 inflammasome recruits ASC, which in turn engages with pro-Cas-1, leading to its activation [194]. The activated Cas-1 then activates pro-inflammatory cytokines IL-1β or IL-18 and GSDMD, thereby releasing the N-terminal GSDMD fragment that forms pores in the plasma membrane. This pore formation induces pyroptotic cell death and facilitates the release of bioactive inflammatory cytokines IL-18 and IL-1β [65,195]. However, NLRP1 detects microbial muramyl dipeptide (MDP), while NLRC4 responds to cytosolic bacteria, and both trigger the Cas-1 activation-mediated inflammatory pathway and subsequent pyroptosis (Figure 1) [196,197].

### 4.2. Non-Canonical Pathway

The word “noncanonical inflammasomes” was first created by Kayagaki et al. [198,199] to elucidate Cas-11 in mice (with the human ortholog caspase 4/5), which is activated via a different mechanism to the canonical NLR-ASC-Cas-1 pattern of activation inflammasome pathway. In the non-canonical pathway, caspases 4, 5, and 11 directly recognize cytosolic LPS released by Gram-negative bacteria [200]. Upon binding to LPS, these caspases cleave GSDMD and produce activated GSDMD, thereby resulting in potassium efflux and the subsequent activation of the inflammasome-mediated inflammatory pathway and cell death. Like the canonical pathway, bioactive GSDMD creates pores in the cell membrane and releases inflammatory proteins, causing pyroptotic cell death [201]. Traditionally, Cas-3 and Cas-8 have been associated with apoptosis, but both caspases can also evoke pyroptosis under certain conditions of the cellular microenvironment and cellular stress. In cells expressing high levels of GasderminE (GSDME), the Caspase-3 cleavage of GSDME results in its activation and subsequent pyroptotic cell death [202,203]. Also, during Yersinia infection, caspase-8 cleaves GSDMD, promoting pyroptosis in murine macrophages [204,205]. Granzymes, such as granzyme B (GzmB) and granzyme A (GzmA), have been found to be connected to the onset of pyroptosis by directly cleaving/activating Gasdermin proteins. Furthermore, GzmB also cleaves GSDME, resulting in the pyroptosis of the target cells. This has a great implication for antitumor responses [206,207]. Similarly to GzmB, GzmA cleaves and activates GSDMB and triggers pyroptotic cell death, thereby causing the reprogramming of tissue homeostasis and cancer continuance [208]. On the whole, it seems that pyroptosis is induced via diverse pathways in inflammasome activation, caspase-mediated Gasdermin cleavage, membrane disruption and inflammatory cytokine release. These mechanisms highlight inflammasome-mediated inflammatory cell death, pyroptosis, as an unquestionable vital event in inflammation and disease pathogenesis (Figure 1).

## 5. NLRP3 Inflammasome Activators, Including Oxidative Stress

Regardless of extensive and thorough investigations to uncover the molecular mechanisms of NLRP3 inflammasome activators of diverse characteristics, they are still an enigma. Herein, we describe some current NLRP3 stimulators that are known to be involved in the activation of NLRP3 inflammasome-mediated inflammatory pathways, resulting in pyroptosis. Potassium (K^+^) efflux is reported to be a prime event elicited in cells during exposure to NLRP3 activators. It has been reported that the diminution of intracellular K^+^ ions is a common phenomenon that facilitates the release of bioactive IL-1β against various NLRP3 activators [105,209]. Also, it is reported that potassium efflux alone can activate the NLRP3 inflammasome pathway, while increased levels of extracellular K^+^ ions can inhibit NLRP3 activation [162,210]. Hence, it is surmised that low intracellular K^+^ levels are a potent activator for the NLRP3 inflammasome [162]. Potassium efflux also regulates calcium-independent phospholipase A_2_ (aiPLA_2_), which helps in IL-1β activation [209]. Calcium plays a pivotal role in cell physiology. However, the role of calcium ions in NLRP3 inflammasome activation is still unclear [209]. Some studies demonstrate that BAPTA-AM (a Ca^2+^ chelator) blocks IL-1β secretion, arguing that calcium is important for NLRP3 inflammasome activation [211,212]. Also, several studies have shown that the NLRP3 activator ATP (particulate matter) and nigericin alter intracellular calcium ion concentrations [213], implicating calcium’s contribution in the NLRP3 inflammasome activation pathway [213,214].

Moreover, potassium (K^+^) and calcium ions (Ca^2+^), chloride ions (Cl^−^) and sodium ions (Na^+^) have also been implicated to perform crucial roles in NLRP3 inflammasome activation. A decrease in chloride ion amounts increases ATP-triggered IL-1β bioactivation and secretion; in contrast, increased extracellular chloride ion concentrations inhibit secretion [215]. Studies have shown that sodium ion influx promotes NLRP3 inflammasome activation, while the inhibition of sodium ion influx by reducing extracellular sodium ion concentrations prevents NLRP3 activation. Reagents like gramicidin, nigericin, and potassium-free media dramatically induce sodium ion influx, thereby reducing potassium ion efflux and activating NLRP3 inflammasome assembly. Recent studies firmly dictate that many NLRP3 inflammasome activators produce ROS, which are essential for inflammasome activation [68,80,216]. In mammalian cells, ROS are generated by enzymes such as NADPH oxidases (NOXs), xanthine oxidases (XOs), cyclooxygenases, and lipoxygenases, in addition to the mitochondrial electron transport chain [217]. The NOX family of transmembrane proteins is particularly implicated in ROS production by NLRP3/NALP3 activators, transferring electrons from cytosolic NADPH to extracellular or luminal O_2_ across biological membranes [218,219]. Previously, it was thought that NLRP3 acts as a cytosolic receptor, but it appears highly unlikely that NLRP3 acts like a receptor to directly interact with all known activators. However, several known activators of the NLRP3 inflammasome are ROS producers, which are shown to be a major cause for NLRP3 inflammasome complex formation and activation in response to several extrinsic and intrinsic stimuli [68,150,216], suggesting that ROS contribute largely to inflammasome activation.

Further, ROS have been observed to activate several tyrosine kinases and G-protein-coupled receptors. The MEK1/2 MAP kinase pathway is activated downstream by Raf kinase, leading to the stimulation of extracellular signal-regulated protein kinases 1 and 2 (ERK1/2) [220]. These are further supported when a suppression in ROS levels correlates with a suppression of ERK1/2 activation and a decrease in the production of pro-inflammatory cytokines, such as TNF-α, IL-1, and IL-18 [221]. The ROS-dependent activation of PI3K and the inflammasome has been established. Recent published findings have highlighted the involvement of the TRX-TXNIP system in the increased production of ROS and ROS-mediated inflammasome activation, depending upon the magnitude of TRX-TXNIP (an antagonist for antioxidant TRX) interaction under the conditions of the cellular microenvironment [193,222]. However, despite convincing evidence supporting the role of ROS in inflammasome activation, there are conflicting reports regarding its precise and authentic role in the activation process of the NLRP3 inflammasome. For example, macrophages deficient in superoxide dismutase-1 (SOD-1) produce higher levels of ROS, but these cells release significantly reduced levels of bioactive IL-1β in response to inflammasome activators [223,224,225]. In contrast to this, our group has recently reported that Prdx6 deficiency in lens epithelial cells displays the increased activation of NLRP3 and secretion of bioactive inflammatory cytokines [6]. In this scenario, we believe that further investigation is required to elucidate the dual role of ROS in the inflammatory pathways and its potential implications for therapeutic interventions in NLRP3 inflammasome-mediated inflammatory diseases. Furthermore, NLRX1, a member of the NLR (nucleotide-binding oligomerization domain-like receptor) family, plays a very different role in ROS generation. Unlike typical NLRs located in the cytoplasm, NLRX1 is linked to the mitochondrial membrane. Structurally similar to the NOD (nucleotide-binding oligomerization domain) subfamily, NLRX1 is implicated in ROS production in response to TNF-α [226]. Previously, it was thought that NADPH oxidase (NOX) is the primary source of ROS. However, studies employing the pharmacological or genetic inhibition of NADPH oxidase have demonstrated no impact of NOX on NLRP3 activation in both murine and human cells [227,228,229]. Instead, cytosolic ROS have been critical in NLRP3 activation [230]. Recently, it has been found that peroxidated lipid accumulation released from damaged cells in the presence of ROS within the cellular microenvironment plays a role in the activation of NLRP3 wherein ARF1 presence was requisite for the NLRP3 activation process [231]. (Table 1).

## 6. NLRP3 Inflammasome Activation and TXNIP

**Figure 3:**
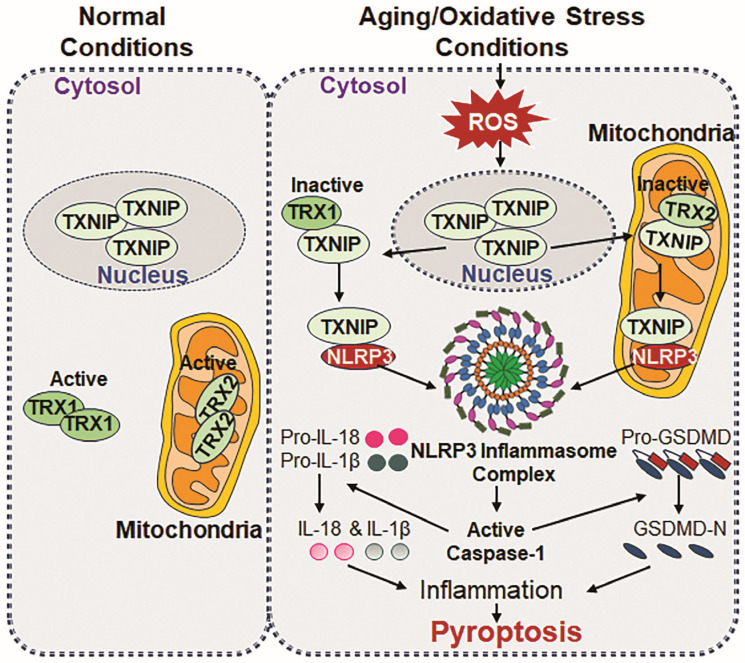
Schematic representation of TXNIP and NLRP3 interaction during aging/oxidative stress. Normal conditions: In normal conditions, TXNIP localizes in the nucleus and plays a role in maintaining cellular physiology. Regarding its binding partners, TRX1 is found in cytosol, while TRX2 localizes in mitochondria, wherein both proteins bind to apoptosis signal-regulating kinase 1 (Ask1), thereby regulating redox homeostasis and ensuring normal cell survival by suppressing excessive ROS accumulation-driven oxidative stress. In aging/oxidative stress conditions: During aging/oxidative stress conditions, TXNIP translocate from the nucleus to cytosol and mitochondria and binds to TRX1 and TRX2, forming TRX1-TXNIP and TRX2-TXNIP complexes, and acts as an inhibitor for TRX activities. The complexes promote Ask1 phosphorylation, leading to the generation and accumulation of cytosolic and mitochondrial ROS, respectively, which results in excessive oxidative stress in the cell. The accumulation of ROS triggers a direct interaction between TXNIP and NLRP3, initiating the formation and activation of NLRP3 inflammasome assembly, including ASC and Cas-1. Active Cas-1 is responsible for the maturation process of IL-1β and IL-18 and mediate their secretion into cytosol, which facilitates inflammatory signaling. At the same occasion, bioactive Cas-1 cleaves GSDMD at the N-terminal domains, which promotes membrane pore formation for the release of IL-1β and IL-18, and the subsequent release of cytokines triggers pyroptosis, a form of inflammatory cell death, contributing to chronic inflammation and age-related pathology and disease state.

Recent reports have highlighted the involvement of the TRX system with its natural inhibitor, TXNIP, in ROS-mediated inflammasome activation [193,222]. TXNIP directly engages the major antioxidant protein TRX and thereby inhibits its antioxidant function and expression [243,244]. TXNIP is a multitasking protein with other important functions beyond modulating intracellular oxidative stress [39,243]. TXNIP can translocalize between diverse intracellular locations, binds to different proteins and has diverse activities under oxidative stress. However, the primary function of TXNIP is implicated to induce apoptosis or pyroptosis, depending upon its oxidative stress status [243]. This molecule is a key regulator of oxidative stress and inflammation. The TXNIP activation of NLRP3 inflammasome signaling is found to be involved in various organ systems such as the nervous, cardiovascular, and respiratory systems and modulates the status of the afore-mentioned systems. Hence, NLRP3-TXNIP interaction is a critical step to understand how their interaction occurs and how and what conditions ROS facilitate that lead to the aberrant activation of the NLRP3 inflammasome inflammatory pathways [245] (Figure 3). Recently, it has been shown that ROS activate the NLRP3 inflammasome and facilitate the interaction between TXNIP and NLRP3 in general [246], but further work is needed to have a clearer understanding. A well-established inflammasome inducer, LPS, also increases ROS accumulation, which leads to the activation expression of NLRP3 and NLRP3 inflammatory genes (ASC, bioactive Cas-1, IL-1β, and IL-18) and TXNIP in BV-2 cells, emphasizing the vital role of ROS in the NLRP3 inflammasome activation pathway. Further, as an antioxidant, NAC treatment has been found to alleviate the interaction between TXNIP and NLRP3 and reverse LPS-driven NLRP3-inflammasome activation [247]. Skeletal muscle fibers from insulin-resistant mice exhibit increased ROS and LPO levels and reveal the aberrant activation of NLRP3 inflammasome with increased ROS-dependent interaction between TXNIP and NLRP3, and this is disrupted by NAC or MCC950 treatment (an NLRP3 inflammasome inhibitor) [246].

TXNIP is also involved in the activation of endoplasmic reticulum (ER) stress-induced NLRP3 inflammasome assembly formation. This activated event has been noticed to elicit mitochondrial stress-induced apoptosis or inflammatory cell death, pyroptosis [39]. Increased levels of TXNIP and NLRP3 in mice with cerebral ischemia/reperfusion injury is implicated to be pivotal in the development of neuroinflammation. It is worth knowing that TXNIP knockdown or its inhibitor(s) holds promise as a therapeutic strategy for cerebral ischemia/reperfusion injury [248]. Chronic intermittent hypoxia (CIH) is an inducer of ROS-driven oxidative stress and inflammation implicated in the etiology of cardiovascular diseases. CIH evokes the TXNIP/NLRP3/IL-1β pathway and mitochondrial dysfunction, while the depletion of TXNIP rescues the endothelial cells from CIH-induced damage [249]. Nonetheless, aging is an inevitable natural process, NLRP3 inflammasome-induced inflammatory response has been reported to play a role in the onset of age-related diseases. Studies have shown that TXNIP regulates NLRP3 inflammasome-induced pyroptosis via cAMP/PKA and P13K/Akt signaling pathways [250]. The work of Cheng et al. has shown that ROS, TXNIP overexpression, and their interaction with NLRP3 may trigger an inflammatory response through the ROS/TXNIP/NLRP3 axis pathway. It has been discerned that E690, E693, and D745 amino acids in NLRP3 and the K212 and R238 amino acids in TXNIP play a key role in TXNIP-NLRP3 interaction [251]. *TXNIP* deficiency impairs the activation of the NLRP3 inflammasome, leading to the inhibition of IL-1β secretion. *TXNIP^−/−^* and *NLRP3^−/−^* mice showed improved glucose tolerance and insulin sensitivity [193]. These studies suggest that using antioxidant therapy to block aberrant inflammasome inflammatory signaling should be a vital strategy.

## 7. Redox-Active Transcription Factors, NF-ĸB and Klf9, and NLRP3 Inflammasome Regulation

**Figure 4:**
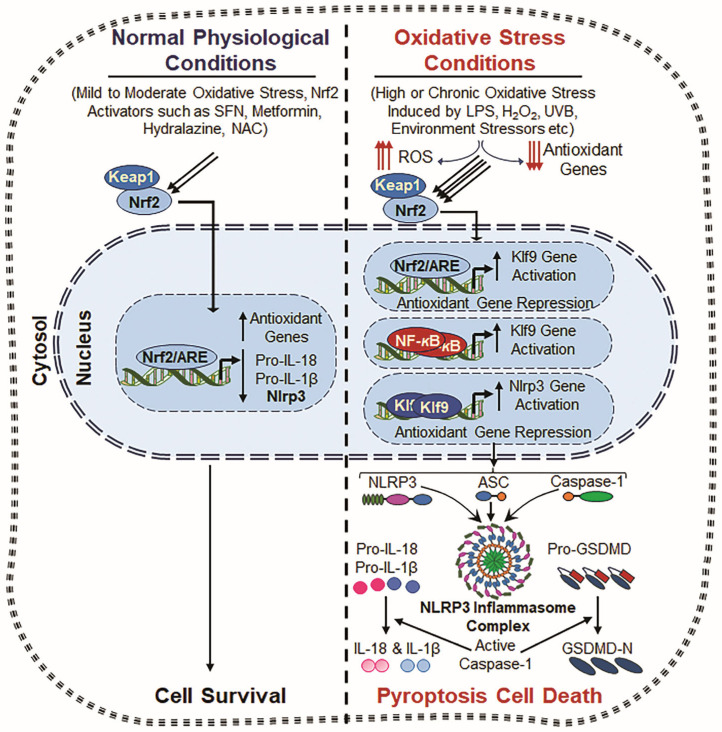
Crosstalk between Nrf2, Klf9, NF-*κ*B and NLRP3. Normal Physiological Conditions: Under normal physiological conditions, Nrf2 remains in the cytoplasm, bound to Keap1 (Kelch-like ECH-associated protein 1), which promotes its degradation via the ubiquitin–proteasome system. However, upon mild to moderate oxidative stress or stimulation by Nrf2 inducers such as Metformin, Hydralazine, and N-acetylcysteine (NAC), Nrf2 is released from Keap1 and translocates to the nucleus. Now, nuclear Nrf2 binds to the antioxidant response element (ARE) present in the promoter regions of various antioxidant genes, including Prdx6, HO-1, NQO1, SOD, and GPx [51,267,271], and enhances the antioxidant defense system and ROS suppression with attenuating NLRP3 inflammasome action. This process results in inflammation prevention and cell toxicity. Oxidative Stress Conditions: During chronic or excessive oxidative stress, induced by factors such as LPS, hydrogen peroxide (H_2_O_2_), ultraviolet B (UVB) radiation, and external or internal stressors, the antioxidant defense system becomes dysregulated, leading to excessive ROS generation accumulation. Under this excessive ROS environment, Nrf2 overly accumulates in the nucleus and induces Klf9 transcription, which paradoxically amplifies ROS production by suppressing antioxidant gene expression [28,51]. Also, NF-*κ*B signaling is activated due to oxidative stress, further enhancing Klf9 transcription. This results in further increased ROS accumulation, creating a positive feedback loop that drives cellular damage and inflammation. Consequently, both Klf9 and NF-*κ*B cooperatively promote increased NLRP3 transcription, leading to the formation of the NLRP3 inflammasome complex, which includes the NLRP3 sensor protein, ASC and Pro-Cas-1. Activated Cas-1 cleaves IL-1β and IL-18 and GSDMD, which leads to the release of bioactive cytokines (IL-1β and IL-18) and pore formation, respectively, and thereby contributes to inflammatory cell death and tissue pathology.

Redox-active transcription factors contribute greatly to the maintenance of cell health, while their aberrant regulation/activation results in cellular derangement, the failure of cellular homeostasis, and cell death. Among all, an important transcription factor, NF-*ĸ*B, is expressed in the cytoplasm in almost all types of cells [6,252]. NF-*ĸ*B is activated by various conditions and stimuli, like oxidative stress, cell pathology, hypoxia, inflammatory mediators, and the internal cellular microenvironment [252]. This transcriptional molecule plays diversified roles, from cell survival to cell death, depending upon the cellular redox environment [242,253]. Based on the effects and mechanisms, the NF-*ĸ*B signaling pathway is classified in the canonical and noncanonical pathways. The canonical pathway is the most studied, wherein NF-*ĸ*B is activated through pro-inflammatory cytokines, including various pathogens and oxidative stress. In normal physiological conditions, inactive NF-*ĸ*B resides in the cytoplasm. Upon activation, NF-*ĸ*B is released from the I*ĸ*B protein and translocates to the nucleus, thereby regulating gene expression involved in cell survival and vice versa. The non-canonical pathway triggered by the TNF receptor superfamily leads to the processing of a precursor protein, p100, into p52, which makes a dimer with RelB. However, both pathways are essential in modulating NF-*ĸ*B expression and activation [254,255]. It is worthwhile to note that aberrant NF-*ĸ*B activation is found to be associated with pro-aging pathways, including NLRP3 inflammasome expression and activation [253,255]. Depending on the cell type and cell environment, NF-*κ*B acts as both proapoptotic and antiapoptotic factors [252,256]. Previously, our group demonstrated increased nuclear NF-*ĸ*B expression with the phosphorylation of I-*ĸ*B (in cytosol) in redox-active *Prdx6*-deficient mLECs [252,257] (Figure 4).

The Kruppel-like factor (Klf) family has 18 members of transcriptional proteins, which are involved in regulating an array of genes of variable functions [258]. Among all, Klf9 is found to be highly expressed in aging cells, including aging eye lenses/lens epithelial cells (LECs) or cells facing oxidative stress [6,28,51]. Tunicamycin (an inducer of cell death)-induced endoplasmic reticulum stress showed the modulation of Nrf2-Klf9 [259]. We have shown that excessive oxidative stress or higher amounts of sulforaphane (SFN) dramatically enhances Klf9 expression via the overaccumulation of Nrf2 in the nucleus. This aberrant activation of Klf9 increased ROS production by suppressing the antioxidant genes [28,51]. Also, aberrant upregulated Klf9 has been observed after myocardial infarction (MI), resulting in inflammatory responses and cell damage. Klf9 promotes Toll-like receptor 2 (TLR2) expression by directly binding to its promoter region and triggers the activation of MAP and NF-*ĸ*B signaling, which contributes to excessive inflammatory responses [50,260]. Importantly, aging LECs or LECs exposed to H_2_O_2_ or LPS, and *Prdx6*-deficient redox-active mLECs display an activated NF-*ĸ*B-Klf9-ROS-NLRP3 inflammasome axis, including bioactive inflammatory components such as ASC, cleaved Cas-1, IL-1β, IL-18, and GSDMD, and the activation of the inflammasome pathway results in pyroptotic cell death [6]. For the innate immune response, the NLRP3 inflammasome and NF-*ĸ*B are critical components and are connected in regulating inflammation. Klf9 also plays a large role in immune responses and cellular differentiation and has also been implicated in modulating various inflammatory pathways [261]. Interestingly, the loss of Klf9 suppresses the inflammatory responses of macrophages triggered by myocardial infarction via the inhibition of NF-*ĸ*B activation and MAPK signaling. Klf9 is a pro-inflammatory transcription factor in macrophages and may be a novel therapeutic target for treating ischemic heart disease [50]. Our group has shown that the overexpression of Klf9 heightened LEC sensitivity to oxidative stress and increased NLRP3 transcriptional activity. In contrast, Klf9-deficient LECs exhibited resistance to H_2_O_2_ or LPS-induced oxidative stress and suppressed oxidative stress-induced NLRP3 transcription [6]. The loss or reduced expression of Prdx6 induces ER stress, leading to the upregulation of ER stress-associated proteins. Oxidative stress triggered by hypoxia, 1% O_2_, cobalt chloride (CoCl_2_), and tunicamycin further amplifies ER stress markers and thereby promotes NLRP3 inflammasome activation in LECs. Interestingly, Prdx6 supplementation alleviates ER stress and NLRP3 inflammasome-driven inflammatory pathways and protects lens cells [6,183].

## 8. Role of Nrf2 and NLRP3 Inflammasome

A master transcription factor, Nrf2, plays a key role in maintaining cellular redox homeostasis in favor of cell health by regulating antioxidant genes [28,48]. Nrf2 also alleviates oxidative stress-induced inflammatory responses by repressing inflammatory genes, contributing to subsiding inflammatory activities [262,263,264]. A deteriorated Nrf2-antioxidant signaling pathway during aging or oxidative stress conditions leads to age-related pathobiology and disorders. Xu et al. found an inhibitory regulation of the Nrf2/ARE pathway on ROS-driven NLRP3 inflammasome activation in BV2 microglial cells following oxygen–glucose deprivation/reoxygenation (OGDR) [264]. It is observed that during the activation of the NLRP3 inflammasome, Nrf2 localizes in the ASC-enriched cytosolic compartment. And *Nrf2*-deficient mice showed a dramatic decrease in immune cell recruitment and bioactive IL-1β release in alum-induced peritonitis [265], pointing out that Nrf2 has great potential for abating inflammatory signaling, if overexpressed or induced by its inducers, like Metformin or sulforaphane. In our recent publication, we have shown that aging lens cells with deteriorated Nrf2-Prdx6 expression and *Prdx6*-deficient LECs contain an activated NLRP3 inflammasome [6,26,46,266]. Sulforaphane, Metformin and Hydralazine enhanced antioxidant gene expression, including Prdx6 via Nrf2 activation [51,263,267,268]. We think that Nrf2 activation should be a therapeutic strategy to control NLRP3 inflammasome-driven inflammatory response. However, the overaccumulation of Nrf2 in the nucleus by its inducers or oxidative stressors like H_2_O_2_ represses Prdx6 transcription, including other major Phase II antioxidant genes by activating Klf9 gene transcription [51]. Furthermore, it is observed that crosstalk between the SIRT1-Nrf2 pathway inhibits NLRP3 inflammasome activation [269]. Also, it has been reported that an Nrf2 activator, Tert-butylhydroquinone (TBHQ), reduces the ROS activation of the NLRP3 inflammasome pathway and IL-1β and IL-18 expression, thereby inhibiting pyroptosis via activating the Nrf2-heme oxygenase 1 (HO-1) signaling pathways [270].

## 9. NLRP3 Inflammasome and Mitochondrial Dysfunction

Earlier studies suggest that NLRP3 recognizes markers of mitochondrial dysfunction/disintegration, including ROS and mtDNA damage and release [150,237,240]. NLRP3 inducers like nigericin cause mitochondrial damage and the release of mtDNA, ROS and oxidative DNA, which directly activates the NLRP3 inflammasome [29]. Several studies have pointed out that NLRP3 adheres to mitochondria during the activation process and that a link is needed for this function [149,177]. It seems that the mitochondrial membrane can serve as a scaffolding site for NLRP3 response to mitochondrial damage. Further, age-related mitochondrial deterioration and dysfunction increase the accumulation of ROS, which may promote NLRP3 inflammasome activation [29,241]. However, the exact contribution of ROS and mitochondrial dysfunction in NLRP3 inflammasome activation is still a matter of debate. Mitochondria are powerhouses; therefore, they are a major source of ROS production, and ROS are optimized via antioxidants present in mitochondria and elsewhere in favor of cell health. Under the conditions of defective mitochondrial respiration, ROS production is progressively increased. This suggests that mitochondrial respiratory chains can be responsible for NLRP3 inflammasome activation [162]. In addition, mitochondria physically co-localize with the NLRP3 inflammasome, with molecules such as cardiolipin, mitofusin 2, and mitochondrial antiviral-signaling protein (MAVS). These molecules have been reported to be involved in the regulation/activation of the NLRP3 inflammasome in response to NLRP3 stimuli [238,239]. Conclusively, ROS originating from various cellular sources, including mitochondria and NOX, play a key role in NLRP3 inflammasome activation across different inflammatory contexts. It is certain that further investigation is required to disclose the molecular mechanisms involved in the regulation of the mitochondrial generation of ROS and its link to NLRP3 inflammasome activation for developing therapeutic strategies targeting inflammasome-related inflammatory pathobiology and disease onset.

## 10. Oxidative Stress, NLRP3 Inflammasome and Aging-Related Disease

In advancing age, the excessive production of ROS overwhelms the antioxidant defense system, which results in oxidative stress-induced cellular damage, including the oxidation of micro/macromolecules of cells [272]. ROS, including superoxide anion and hydrogen peroxide, are highly reactive reagents that can inflict enormous oxidative damage on cellular components like proteins, DNA, and lipids, contributing to various pathologies [273,274,275]. Studies indicate that increased ROS accumulation due to the dysregulation of antioxidant response plays a pivotal role in reprogramming signaling cascade(s) that lead to cellular injury and death [276,277,278,279]. Moreover, major hallmarks of aging-related diseases, including aging-related cataract, is the inability of antioxidant responses to act against oxidative stress due to the deterioration/dysregulation of cellular antioxidant system. Importantly, it is now known that oxidative and aging-related pathologies share a series of common denominators, and studies suggest that delaying/treating one oxidative/age-related disorder can mitigate the progression of others [6,62,280,281,282,283,284]. These studies emphasize that a supply of antioxidants can prevent the progression or initiation of inflammatory disorders associated with aging and oxidative stress. Furthermore, ROS accumulation is shown to be involved in the development of age-related diseases regardless of origin. Over the decades, many theories on aging have been proposed and some have been corroborated by advances in molecular biology [285]. Denham Harman’s Free Radical Theory of Aging in 1956 postulated that oxygen-derived free radicals (produced as byproducts of metabolism) can be a plausible cause of oxidative DNA/protein damage, serving as activators of the NLRP3 inflammasome inflammatory pathway, leading to sterile inflammation and “inflammaging”-related disease onset [17,62,81,286]. Recent accumulating research revealed a more nuanced perspective, suggesting that moderate levels of oxidative stress can be beneficial, acting as important survival signaling [28,48,51]. For instance, an antioxidant enzyme, SIRT1, plays a pivotal role in enhancing antioxidant production via the FoxO pathway, thereby regulating oxidative stress. In contrast, excessive ROS can impair SIRT1 function by oxidizing its cysteine residues, activating NF-*ĸ*B signaling, a key driver of inflammatory responses and inflammasome activation [287]. Moreover, the naturally occurring inherited immune system utilizes pattern recognition receptors like TLRs and NLRs. NLRs selectively detect cellular stress, thereby eliciting inflammasome activation, which subsequently mediates the maturation and secretion of cytokines IL-1β and IL-18 [58,288]. Oxidative stress and disruptions in thiol-redox balance have also been shown to activate inflammasomes in recently published studies [53,221].

The NLRP3 inflammasome inflammatory pathway has been observed to be involved in a wide spectrum of oxidative-or age-related disorders, such as autoimmune diseases, type-2 diabetes, neurological disease, ocular disease, age-related hearing loss, cancer, metabolic syndrome, dementia, including vascular and frontotemporal dementia, and so on [78,289,290,291,292,293,294,295]. In neurological diseases such as Alzheimer’s disease (AD) and Parkinson’s disease (PD), including other tauopathies, the activation of the NLRP3 inflammasome is triggered by protein aggregates, such as β-amyloid plaques and tau protein fibrils (protein aggregates). Interestingly, recently, β-hydroxybutyrate administration has been found to abate AD pathobiology with a reduction in plaque formation by inhibiting NLRP3 inflammasome activation [295]. Like other neurological disorders, amyotrophic lateral sclerosis (ALS) involves the NLRP3 inflammasome activation-mediated inflammatory pathway in response to pathogenic proteins, such as β-amyloid and α-synuclein. This activation results in Cas-1 activation and IL-1β release, and promotes ALS progression [296]. Moreover, cardiovascular diseases have also been identified to be associated with the aberrant activation of NLRP3 inflammasome and oxidative stress. Nonetheless, despite promising evidence showing the involvement of inflammation, the majority of anti-inflammatory drugs used in clinical trials failed to prevent cardiac arrest [297]. Many other cardiovascular disorders, like structural cardiomyopathy and idiopathic cardiomyopathy, are also linked to the NLRP3 inflammasome activation-mediated inflammatory pathway [78]. Furthermore, NLRP3 activated by islet amyloid polypeptide aggregates has been implicated to be associated with type 2- diabetes [298]. Activated NLRP3 inflammasomes and inflammation have been detected in blinding diseases and the role of NLRP3 in the etiology and development of age-related eye diseases such as age-related macular degeneration (AMD), diabetic retinopathy, glaucoma and other chronic ocular diseases has been established [67,299,300,301]. Recently, the NLRP3 inflammasome is recognized as a major cause of the pathogenesis and progression of AMD. Furthermore, studies have shown that increased levels of blood glucose and oxidative stress along with chronic inflammation are the prime factors for diabetic retinopathy. This process leads to immune cell infiltration and an elevation in pro-inflammatory cytokine release with NLRP3 upregulation [299,301]. Excessive uric acid is associated with age-related cataract formation, and the increased expression of NLRP3, Cas-1 and senescence regulators p53 and p21 was noticed in the lens capsules of a hyper-uricemic patient [302]. It is worthwhile to emphasize that dry eye disease is a chronic ocular surface disorder wherein the NLRP3 inflammasome inflammatory pathway has been implicated as a key driver for ocular surface inflammation [303]. Activated NLRP3 with increased Cas-1 and ASC expression with increased pro-inflammatory cytokines such as Il-1β and IL-18 were observed in aging lens/lens epithelial cells (LECs) and LECs facing oxidative stress [6]. It has been recently shown that inflammatory cell death, pyroptosis, is a cause for cataract formation [302,304,305] and corneal inflammation [306]. Sensorineural hearing loss is the most common type of hearing loss in adults. Recently, research has provided evidence that NLRP3 inflammasome-driven activated macrophages are involved in hearing loss [307]. Collectively, these studies underscore the pivotal role of NLRP inflammasome activation for the development of several diseases, thereby warranting further exploration as a therapeutic target.

## 11. Potential Therapeutics Against Inflammasome Dysregulation and Inflammation

During aging or oxidative stress, the aberrant NLRP3 inflammasome activation-driven inflammatory pathway has been causally related to the development of aging-related diseases [179,308]. Since the NLRP3 inflammasome has been identified as a research hotspot, strategic research efforts have been made in the identification of NLRP3 inflammasome-targeting small molecules or chemical agents to use against NLRP3 inflammasome activation. In this regard, several specific inhibitors of NLRP3 have been reported; only some of them have been clinically tested [293,294,295,308,309]. These NLRP3 antagonists have better potential for further evaluation and study and even for clinical treatment. Several studies have shown that the sulfonylurea compound MCC950 is currently the most recognized and representative NLRP3 inflammasome inhibitor since being discovered and reported [310]. The application of this small molecule blocks canonical and noncanonical NLRP3 activation, even at lower concentrations like nanomolar concentrations. MCC950 is a specific inhibitor of the NLRP3 inflammasome, but this reagent is not effective in the case of other inflammasomes such as AIM2, NLRC4 or NLRP1. MCC950 prevents the release of pro-inflammatory cytokines like IL-1β and IL-18 by binding to the NLRP3 protein [310]. Several investigators have tested its potential to treat various diseases connected to the NLRP3 inflammasome, such as diabetes, retinal endothelial cell dysfunction, heart muscle damage, osteoarthritis and AD [311]. MCC950 has been found to treat a variety of disorders but, unfortunately, failed to pass in Phase II clinical trials of rheumatoid arthritis due to its hepatotoxicity [309]. However, some investigators have modified its structure to reduce its hepatotoxicity and found better NLRP3 inhibitors, such as ZYIL1 [312,313]. We believe that MCC950 derivatives could have a potential therapeutic effect in treating various disorders related to NLRP3 inflammasome activation. ZYIL1, a derivative of MCC950, is a novel specific inhibitor that has been discovered. In Phase II clinical trials of CAPS (Cryopyrin-Related Cycle Syndrome) patients conducted by Zydus Company, this small molecule showed good efficacy. Compound CY-09 is also known to specifically block NLRP3 inflammasome activation by interacting with the ATP-binding motif of the NLRP3 NACHT domain and inhibit NLRP3 ATPase activity. CY-09 application has been found to be very effective to blunt cryopyrin-associated autoinflammatory syndrome and type 2 diabetes [314]. Recently the drug Oridonin has been found to have anti-inflammatory properties, and therefore, this can serve as a lead for developing new therapeutics against NLRP3-associated diseases such as peritonitis, gouty arthritis and type 2 diabetes. Oridonin inhibits NLRP3 inflammasome assembly and activation by binding to the cysteine 279 of NLRP3 in the NACHT domain to block NLRP3 and NEK7 interaction [315]. Currently, Union Medical Hospital is carrying out clinical trials of CY09 to treat NLRP3 inflammasome activation-mediated coronary artery disease (CAD). Recently, a small molecule, DFV890, that inhibits NLRP3 selectively has been under clinical trials. Novartis conducted an early randomized clinical trial of this molecule in patients suffering from COVID-19 pneumonia and respiratory syndrome and found an encouraging result in clearance with improved clinical status [316]. Furthermore, studies have shown that BHB (β-hydroxybutyrate) abates IL-1β and IL-18 production by blocking the NLRP3 inflammasome [317]. However, currently, there are no clinical trials for this compound for NLRP3-related disorders, but it has been suggested to test the efficacy of BHB in clinical trials. Moreover, an old anti-allergic drug, Tranilast, has been shown to inhibit NLRP3 inflammasome activation. Tranilast attenuates NLRP3 activation by interfering with NLRP3 oligomerization as it directly binds to the NACHT domain of NLRP3. Like Compound CY-09, Tranilast could be a potential pharmacological approach for the prevention/treatment of NLRP3-driven diseases such as type 2 diabetes, gouty arthritis, and cryopyrin-associated auto-inflammatory syndrome [318]. Clinical trial drugs for NLRP3 inhibitors such as Dapansutrile, Selnoflast, emlenoflast, NT0796, VTX2735, VTX3232ZYIL1, an agonist (BMS-986299), and an antagonist (IFM-2427) have been reported [72,319,320]. Furthermore, in deep venous thrombosis rats, TRX expression was suppressed while TXNIP and NLRP3 were elevated, wherein siTXNIP or MCC950 injection rescued the injury of a vein induced by deep venous thrombosis by improving the physiological changes and reducing the inflammatory reaction [321]. On the whole, studies have apparently shown that the NLRP3 inflammasome is a very promising therapeutic target, but unfortunately, there is no approved NLRP3 inhibitor(s) in the market to date. However, clinically, only IL-1β antibodies can be employed to treat NLRP3-associated disorders. This treatment is evidently insufficient as IL-18 is also involved in NLRP3 inflammasome-mediated inflammatory cell death. Thus, the discovery of NLRP3 inhibitors (s) is warranted for the treatment of NLRP3 inflammasome-mediated pathobiology and its associated aging diseases. (Table 2).

## 12. Future Direction and Conclusions

In recent studies, NLRP3 activation and its crucial role in immune responses and non-immune systems and the release of pro-inflammatory cytokines has been recognized widely. NLRP3 activation is triggered by various stimuli, such as aging, ROS, and mitochondrial dysfunction, as noted in the above sections. However, the intrinsic relationship and molecular mechanisms involved in NLRP3 inflammasome activation, oxidative stress, aging, and ocular diseases are critical areas of investigation. Age-related pathologies worsen due to oxidative stress, and its contribution to the onset of ocular diseases such as cataract, glaucoma, AMD, diabetic retinopathy is well established but requires unveiling the molecular mechanism(s) involved to develop therapeutic agents. Also, a future goal should be identifying molecular triggers of NLRP3 inflammasome activation in aging eye lenses, retinas, corneas, and trabecular meshwork cells, including other organs. We think that the identification of the culprit factors should be used for developing targeted therapies against NLRP3 inflammasome activation-driven disorders. In addition, we would like to reconsider investigating deeper the role of the TXNIP and oxidative stress axis in NLRP3 activation in the eye. We anticipate the need to determine the functional role of Klf9 in mitochondrial oxidative damage and its impact on cellular aging and the type of cell death, like apoptosis, pyroptosis, necroptosis, panoptosis, and ferroptosis, and crosstalk among these different types of cell death. This will help to discover a novel therapeutic reagent that can block all types of cell death. We know that NF-*ĸ*B plays a critical role in the expression and activation of inflammation; how NF-*ĸ*B mediates inflammation linked to TXNIP and Klf9 to drive disease progression in an aging eye needs to be explored. In this scenario, currently, several target genes have been investigated that are involved in the aberrant regulation and activation of NLRP3 inflammasome-mediated inflammation and pathology; therefore, future research should identify and explore novel inhibitors against the prime target molecules involved in NLRP3 inflammasome signaling. Furthermore, pro-inflammatory cytokines are released upon NLRP3 activation. So future research is needed to develop the inhibitors of downstream inflammatory cytokines. We also need to develop an age-specific strategy to prevent inappropriate NLRP3 activation in various age-related diseases. We also need to develop gene therapy approaches, such as the CRISPR-based modulation of Klf9 or TXNIP (involved in the dysregulation of ROS homeostasis, as noted above) to restore antioxidant balance in aging ocular tissues and other organs/tissues. To identify potential intervention points, we need to investigate and reinvestigate the interaction between oxidative stress and TXNIP-Klf9-NF-*ĸ*B and their contribution(s) in the dysregulation of the Nrf2 antioxidant system. Furthermore, we should explore how age-related epigenetic changes influence antioxidant defense and inflammatory responses and whether epigenetic modulation could serve as a treatment strategy. Finally, we think that efforts should be made to evaluate and identify the therapeutic benefits of dietary and pharmacological antioxidants in suppressing aberrant TXNIP-NLRP3 expression and inflammasome activation signaling to delay or prevent age-related ocular diseases and other disorders in general.

In conclusion, consolidating the knowledge and pathway analysis of the NLRP3 inflammasome and its inflammatory components, theTXNIP-Klf9-NF-*ĸ*B axis, and oxidative stress in aging/oxidative-linked ocular diseases, including other diseases, will pave the way to develop precision medicine approaches. Also, a deeper understanding of the above-noted biological molecules and their pathways and activities will facilitate the development of target factor-based therapeutics and approaches aimed at preserving vision and preventing age-related blindness and other NLRP3 inflammasome-linked disorders in general.

## Figures and Tables

**Table 1:** NLRP3 inflammasome activators.

NLRP3 Inflammasome Activators	Mechanism of Action	References
Lipopolysaccharide (LPS)	pIRAK-1 → NLRP3- inflammasome activation; → Caspase-1 cleavage → release of bioactive cytokines Klf9/NF-ĸB → NLRP3 inflammasome activation → Caspase-1 cleavage → release of bioactive cytokines	[6,17,170,177,178]
Nigericin	Direct activation of NLRP3 inflammasome activation → Caspase-1 cleavage → release of bioactive cytokines	[162,177]
Extracellular ATP	NLRP3 activation → facilitates release of bioactive cytokines; K^+^ efflux and NF-ĸB priming	[162,176,177,178]
PAMPs, DAMPs, potassium (K^+^), calcium ion (Ca^2+^), chloride ion (Cl^−^)	NLRP3 activation → Caspase-1 cleavage → release of bioactive cytokines	[105,162,177,188,209,210,211,212,215]
Amyloid -β (Aβ)	Activation of Syk and AMPK inhibition → NLRP3 activation in microglia; TLR4 mediated → NLRP3 activation in microglia; cathepsin B release → NLRP3 activation	[232,233]
Reactive oxygen species inducers (H_2_O_2_, UVB, Paraquat, etc.)	Klf9/NF-ĸB → NLRP3 activation in LECs; activates tyrosine kinase and G-protein; activates MEK1/2-MAPK pathways; P13K activation; TXNIP, Klf9 and NF-ĸB activation; ionic efflux → NLRP3 activation	[6,68,80,216,217,218,219,221,222,224,225,226,227,228,229,230,231,234]
Crystal structure (uric acid, cholesterol, silica, asbestos, viral protein and dsRNA), monosodium urate (MSU) crystal	NLRP3 activation via sensors Rif-I and Mda5 and common adaptor Mavs; NLRP3 activation → Caspase-1 cleavage → release of bioactive cytokines	[162,177]
Mitochondrial DNA (mtDNA)	Activation of IRF1 → NLRP3 activation; oxidative stress or age-related mitochondrial dysfunction directly activate NLRP3 inflammasome	[29,149,150,162,172,176,235,236,237,238,239,240,241]
Bacterial RNA, viruses, and fungal infections	NLRP3 activation → Caspase-1 cleavage → release of bioactive cytokines; NF-ĸB → NLRP3 activation and P2X7R and TLR signaling	[162,174,178,226,242]

**Table 2:** NLRP3 inflammasome inhibitors.

NLRP3 Inflammasome Inhibitors	Mechanism of Action	References
MCC950	Blocks canonical and non-canonical pathways of NLRP3, but failed in Phase II clinical trials of rheumatoid arthritis and discontinued due to hepatotoxicity	[293,294,295,308,309,310,312]
CY-09	NLRP3 inhibitor; inhibits NLRP3 ATPase activity and prevents cryopyrin-associated autoinflammatory syndrome and type-2 diabetes; clinical trial conducted to treat NLRP3 inflammasome activation-mediated coronary artery disease (CAD)	[314,322]
DFV890	Selectively inhibits NLRP3 under a clinical trial	[316,323]
ZYIL1	NLRP3 specific inhibitor, Phase II clinical trial of CAPS (Cryopyrin-Related Cycle Syndrome)	[4,312]
Oridonin	Blocks NLRP3 inflammasome activation by binding to Cysteine 279 to block NLRP3-NEK7 interaction → therapeutics target against peritonitis, gouty arthritis, and type-2 diabetes	[315,324]
Tranilast	NLRP3 inhibitor, an anti-allergic drug → attenuates NLRP3 activation by interfering with its oligomerization → type-2 diabetes, gouty arthritis and cryopyrin-associated autoinflammatory syndrome	[318,325]
Dapansutrile (OLT1177); Selnoflast; Emlenoflast; NT0796; VTX2735; VTX3232ZYIL1; BMS-986299 (an agonist); IFM-2427 (an antagonist)	Clinical trial reagents: NLRP3 inflammasome inhibitors under clinical trial	[72,319,320]
β-hydroxybutyrate (BHB)	Abates IL-1β and IL-18 production by blocking NLRP3 inflammasome	[317,326]
Metformin, NAC	Inhibit inflammasome activation by activating Nrf2-AMPK → protect LECs/lens by detoxifying ROS; inhibition of elevated ROS in Cardiocyte aging	[6,17,247,262]
siTXNIP or its inhibitor	Prevents IL-1β and IL-18 release by blocking NLRP3 inflammasome activation	[37,244,246,247,248,250]

## Data Availability

Not applicable.

## Abbreviations

The following abbreviations are used in this manuscript:

NLRP3	NOD, LRR- and Pyrin domain-containing protein 3
ASC	Apoptosis-associated speck-like protein containing a CARD
Caspase-1	Cysteine-Dependent Aspartate-Specific Proteases-1
IL-1β	Interleukin-1 beta
IL-18	Interleukin-18
GSDMD	GasderminD
TLRs	Toll-like receptors
ADP	Adenosine Diphosphate
ATP	Adenosine Triphosphate
PAMPs	Pathogen-associated molecular patterns
DAMPs	Danger-associated molecular patterns
ROS	Reactive oxygen species
NADPH	Nicotinamide Adenine Dinucleotide Phosphate
Nrf2	Nuclear factor erythroid 2-related factor 2
SOD	Superoxide dismutase
GPxs	Glutathione peroxidases
TRX	Thioredoxin
CAT	Catalase
Prdxs	Peroxiredoxins
Prdx6	Peroxiredoxin 6
TXNIP	Thioredoxin interacting protein
NF-κB	Nuclear Factor Kappa-light-chain-enhancer of activated B cells
AP-1	Activator Protein 1
MAPK	Mitogen-activated protein kinases
Klf9	Kruppel-like factor 9
PRRs	Pattern recognition receptors
NLR	Nucleotide-binding domain leucine-rich repeat receptor
RIG-I	Retinoic acid-inducible gene I
CLRs	C-type lectin receptors
NACHT domain	N-terminus, a nucleotide-binding and oligomerization domain
LRRs	Leucine-rich repeats
NLRP1	NLR family Pyrin domain-containing 1
CARD	Caspase activation and recruitment domain
PYD	Pyrin domain
PYPAF5	PYRIN-containing Apaf-1-like protein 5
T3SS	Type III section system
IPAF	ICE protease-activating factor
NAIP	Neuronal apoptosis inhibitory protein
TNFα	Tumor necrosis factor-alpha
AIM2	Absent in melanoma 2
dsDNA	Double-stranded DNA
ALR	AIM2-like receptor
IFI16	Interferon gamma-inducible protein 16
HIN domain/motif	Hematopoietic interferon-inducible nuclear domain/motif
KSHV	Kaposi’s Sarcoma-associated herpesvirus
IFN-γ	Interferon gamma
LPS	Lipopolysaccharide
FMF	Familial Mediterranean fever
RD	Repressor domain
PFD	Pore-forming domain
NLRPs	Nod-like receptors
MDP	Microbial muramyl dipeptide
GSDME	Gasdermin E
GzmB	Granzyme B
GzmA	Granzyme A
PKR	Protein Kinase R
AMD	Age-related macular degeneration
LECs	Lens epithelial cells
K^+^	Potassium
Ca^2+^	Calcium ion
Cl^−^	Chloride ion
Na^+^	Sodium ion
O_2_	Oxygen
NOXs	NADPH oxidases
XO	Xanthine oxidase
TRX	Thioredoxin
ERK1/2	Extracellular Signal-Regulated protein Kinases 1 and 2
NOD	Nucleotide-binding oligomerization domain
NLR	Nucleotide-binding oligomerization domain-like receptor
NOX	NADPH oxidase
MAVS	Mitochondrial antiviral-signaling protein
SFN	Sulforaphane
Klfs	Kruppel-like factors
TLR2	Toll-like receptor 2
CoCl_2_	Cobalt chloride
Nrf2	Nuclear factor erythroid-derived 2-related factor
ARE	Antioxidant response element
OGDR	Oxygen–glucose deprivation/reoxygenation
TBHQ	Tert-butylhydroquinone
HO-1	Heme Oxygenase 1
ER	Endoplasmic reticulum
CIH	Chronic intermittent hypoxia
H_2_O_2_	Hydrogen peroxide
UVB	Ultraviolet B
LPS	Lipopolysaccharide
NAC	N-acetylcysteine
Keap1	Kelch-like ECH-associated protein 1
Ask1	Apoptosis signal-regulating kinase 1
AD	Alzheimer’s disease
PD	Parkinsons disease
CAD	Coronary artery disease
BHB	β-hydroxybutyrate
CAPS	Cryopyrin-Related Cycle Syndrome
